# Evaluation of vestibular evoked myogenic potentials in migraine and migrainous vertigo patients

**DOI:** 10.1186/1129-2377-14-S1-P100

**Published:** 2013-02-21

**Authors:** B ÇatýkerÜtkür, A Güneri, F Ýdiman

**Affiliations:** 1 Dokuz Eylül University, Turkey

## Objective

Studying subclinic vestibular exposure in migraine patients with and without headache, pathophysiology of vertigo in migrainous vertigo patients, electrophysiological similarity and difference between these patients by applying VEMP test to migraine and migrainous vertigo patients.

## Method

26 migrainous vertigo and 22 migraine patients, and 27 healthy controls were enrolled in the study.VEMPtest was applied on ICS-CHARTER auditory potential audiometry.To induce headache by visual stimulation(VS) was used a computer simulation system.VEMP responses were recorded separately before and after VS. Findings:VEMPs were obtained from all the cases in the control group while they could not be obtained from 9 ears in migraine and 5 ears from migrainous vertigo group.There are no significant differences for latent periods of VEMP between migrainous vertigo and control group.The lower VEMP’s amplitudes, higher amplitude ratio and higher threshold of stimulus intensity were found in migrainous vertigo than the controls.The lower interpeak amplitudes and the longer latencies for VEMP were found in migraine compared to controls.The interpeak amplitude value and latency periods were found to be shorter in the migraine compared to the controls.In migrainous vertigo interpeak amplitude value was higher, and the p13 and n23 latency periods were longer than these in migraine.No statistically significant difference was observed between pre and post-headache stimulation VEMP records of migraine.

## Conclusion

The findings of migrainous vertigo were considered in favor of asymmetric peripheral vestibular exposure.The results have also suggested that migraine have subclinic central or mix type vestibuler disorder.On the other hand unchanged VEMP’ findings before and during headache induced by VS led to thinking that vestibular symptoms and headache have different pathogenesis in migraine and migrainous vertigo.In addition shorter latency potentials in migraine have suggested that they may be from results of cortical hiperexitability in migraine.
